# The experience of palliative patients and their families of a family meeting utilised as an instrument for spiritual and psychosocial care: A qualitative study

**DOI:** 10.1186/1472-684X-10-7

**Published:** 2011-03-24

**Authors:** Heather M Tan, Anne Wilson, Ian Olver, Christopher Barton

**Affiliations:** 1 Palliative Care Research Team, School of Nursing and Midwifery, Monash University, Frankston, Victoria 3199, Australia; 2 School of Nursing, University of Adelaide, Adelaide, Australia; 3 Chief Executive Officer, Cancer Council Australia, Sydney, Australia; 4 Flinders Prevention, Promotion and Primary Health Care, General Practice; School of Medicine; Flinders University, Adelaide, Australia

## Abstract

**Background:**

This study explores the experience of palliative patients and their family members of a family meeting model, utilised as an instrument for the provision of spiritual and psychosocial care. In doing so the study embraces a broad understanding of spirituality which may or may not include formal religious practice and a concept of psychosocial care that includes: social and emotional well-being, communication, self esteem, mental health and adaptation to illness. The meeting of spiritual and psychosocial needs is considered to be an important aspect of palliative care.

**Methods:**

This qualitative study, philosophically underpinned by hermeneutic phenomenology, investigates the participatory experience of palliative care patients and their significant family members of such a family meeting. People registered with two large metropolitan palliative care services, who met selection criteria, were referred by medical staff. Twelve of the 66 referred took part in family meetings which also included significant others invited by the patient. A total of 36 family members participated. The number of participants of individual family meetings ranged from two to eleven. After the family meeting every participant was invited to take part in an individual in-depth interview about their experience of the meeting. Forty seven interviews were conducted. These were audio recorded and transcribed.

**Results:**

Data analysis, utilising Ricoeur's theory of interpretation, revealed seven main themes: personal experience of the meeting, personal outcomes, observation of others' experience, observation of experience and outcomes for the family unit, meeting facilitation, how it could have been different and general applicability of the family meeting. Throughout these themes were numerous references to aspects of the web of relationships which describe the concept of spirituality as it is defined for the purpose of this study.

**Conclusions:**

The findings indicate the potential of the type of family meeting reported for use in the spiritual and psychosocial care of people receiving palliative care and their families. However further research is needed to explore its application to more culturally diverse groups and its longer term impact on family members.

## Background

### The importance of spiritual care

It is widely accepted that spiritual care of palliative patients, near the end-of-life, is an important part of their total care and that its provision is a multi-disciplinary task [1-4]. A review of qualitative literature on perspectives on spirituality at the end of life, concluded that the "fundamental importance of spirituality at end-of-life' had been confirmed [5 p.407]. Palliative Care Australia has also listed spiritual care as a key area for research in palliative care [6].

### Defining spirituality

Some literature has differentiated between spirituality and religiosity [7-9] although the drawbacks of such an approach have been discussed [10]. It has been demonstrated that religious well-being and spiritual well-being were conceptually and statistically distinct and had different impacts on health related quality of life [11]. A negative correlation between spiritual well-being and anxiety and depression has been identified but no such correlation was observed in relation to religiosity [12]. A thematic review of literature relating to spirituality in palliative care suggested that spirituality was emerging as a concept largely devoid of religion [13].

On the other hand it has been argued that religion (or faith) is one of three domains of spirituality, along with peace and meaning, rather than being differentiated from it and this premise forms the basis of the Functional Assessment of Chronic Illness Therapy - Spiritual Well-being Scale (FACIT-Sp), used to measure the religious/spiritual components of quality of life [14]. The different component domains of spirituality described in FACIT-Sp have been shown to correlate with quality of life differently [14,15]. The problems of definition and of outcome measures still remain despite a reported increase of 600% in publications relating to the role of religion and spirituality in health in the period 1993-2002 [16].

For the purposes of the current study, the concept of spirituality is described "as the web of relationships that gives coherence to our lives. Religious belief may or may not be a part of that web" [17 pS11]. This web of relationships may include relationships with places, things, ourselves, significant others, and with a power beyond ourselves and is integral to our capacity to find meaning and purpose in our experiences [17-20].

With a spirituality which does not necessarily include adherence to religious doctrine or practice there is a risk that both definitions of spirituality and spiritual well-being measures tap issues such as social well-being and mental health, also described as psychosocial care [21]. Within the palliative care context psychosocial care has been defined as 'concern with the psychological and emotional well-being of the patient and their family/carers, including issues of self-esteem, insight into an adaption to illness and its consequences, communication, social functioning and relationships' [22]. A web of relationships which gives coherence to our lives and is integral to the process of meaning making will inevitably overlap with psychosocial issues, relationships being important in both domains.

### Work with individuals or with families?

There is increasing evidence that working with the whole family rather than focusing only on the patient, especially in a palliative care setting, has better outcomes for both patient and family members [23-25]. The premise that family members and carers' needs must also be addressed is based on the notion that families are systems and that the illness or death of one member alters the balance of the system, which impacts on all other parts of it and requires the negotiation of a new balance [26,27].

A family is conventionally defined as a group of persons linked by kinship, either through blood lines or by marriage [23]. Changing social circumstances have resulted in broader definitions of the family which include those closest to the patient in knowledge, care and affection, some of whom may not be related to the patient by bloodline or marriage [28]. A review of literature relating to working with families in end of life situations indicates that while comprehensive palliative care must involve working with the family unit, numerous dynamics operating in family systems mean that one way of assessing needs and meeting them will not be appropriate for all families [29].

### Working with families in Palliative Care

Family meetings have been promoted in palliative care as important tools for information sharing and care planning and have been shown to be effective in meeting the needs of family carers in particular [30]. The need for health professionals to have skills in communication in order to effectively conduct family meetings has been recognised [31,32] and attempts have been made to address this need with the development of training modules [31]. A literature review did not identify any examples of a family meeting being used solely to address spiritual and psychosocial issues although its use to address potential bereavement issues for families identified as at risk of complex grief has been reported [23].

### Murphy's family meeting model

One way of working with family groups in providing psycho-social and spiritual care has been described by Murphy [32]. This type of family meeting is in many ways a sacred event, a time for making peace, discharging old resentments, giving thanks and saying goodbye. Murphy has developed a five-part paradigm to guide families through this process which includes: the story of the wound (told by the dying member), worries and fears, roots (bringing out memories from the shadows), hearing from other family members, and a blessing or closing of the meeting [32]. One of the fundamental premises of this model is the demonstrated value of telling and re-framing of stories [32-34]. Three main roles are described within the model which are: the story teller (the patient and then other members of the family), the witnesses (those who listen to the story), and the guide or facilitator who has the task of encouraging and supporting the story teller and other family members.

The current study investigated the experience of palliative care patients and their family members, of taking part in a family meeting conducted according to Murphy's model, for the purpose of facilitating spiritual and psychosocial care.

## Methods

The philosophical underpinning of this qualitative study is Hermeneutic Phenomenology [35-37]. Hermeneutic phenomenology, as a process of interpreting and describing human experience in order to understand the central nature of that experience, is a suitable methodology for human sciences research. Increasingly, it is the philosophical underpinning of choice in qualitative health research [38] and has been utilized in a number of disciplines within health services. It was chosen for this study because: it was considered most suitable for the type of investigation being undertaken; of its record of use in qualitative research and because its' understanding of how human beings acquire knowledge and the impact of this in researcher/participant interaction was consistent with that of the researchers in this study. The existence of various forms of phenomenology does, however, create a mine field for the unwary researcher.

Both 'hermeneutics' and 'phenomenology' have been variously defined, but for the purposes of this study they were taken to have the following meanings:

Hermeneutics is the "art and science of interpretation" especially as it applies to text [39].

Phenomenology is the study of the essence of a phenomenon as it presents itself in lived experience in the world [40].

The development of hermeneutic phenomenology was an evolutionary process to which a number of renowned philosophers contributed. Husserl, widely acknowledged as the founder of phenomenology, introduced the term 'life world' which was understood as being what is experienced pre-reflectively [41]. A key to Husserlian phenomenology is a process of bracketing or separating the researchers own thoughts, views etc totally from those of the researched resulting it is claimed in a totally objective view of the data [41]. Duthy's work focused around hermeneutics and he promoted the notion that in gaining understanding we move from the text to the historical and social context of the author and back [37]. This of course has connotations of the hermeneutic circle. Heidegger embarked on a phenomenology of human being, or as he called it Dasein, a term denoting the essential nature of the human being, which includes the ability to enquire into the nature and possibilities of 'Being' [36 p.27]. In his view pre-understanding is a fact of our being in the world and it is not something we can eliminate, or bracket as Husserl claimed. From Gadamer's perspective the interaction between researcher and participant, or between reader and text, is a constant discourse, and hence interpretation is a collaborative process [35]. He sees this process of being one of constant mediation between the past (tradition, culture, experience) and the present 'horizon' (the immediate experience) of the interpreter. Ricoeur, more than any other cemented the connection between hermeneutics and phenomenology and as Thompson points out, the mutual affinity between hermeneutics and phenomenology provided the philosophical basis for much of his work [37, p.21]. He is probably best known for his theory of interpretation which was utilized in the analysis of data in this study. This is discussed in more detail in another publication [42].

Approval to conduct the study was given by the following human research ethics committees: University of Adelaide, Royal Adelaide Hospital, Calvary Health Care Adelaide and The Queen Elizabeth Hospital.

### Recruiting

People receiving palliative care were recruited from among those registered with two metropolitan palliative care services. In addition to caring for patients admitted to two large teaching hospitals and associated hospice facilities, both services also offered an extensive community outreach service. The following selection criteria were applied:

• Patients considered able, physically and mentally, by the attending medical staff, to be present at and participate in the family meeting;

• Patients who were aware of the terminal nature of their illness and were not expected by medical staff to live longer than six months;

• Patients over the age of 18 who were able to converse and give informed consent in English.

Ethics approval required that patients be referred to the researcher by medical or nursing staff.

Those patients who agreed to take part were asked to invite the people important to them to attend the family meeting. These family members were over the age of 16 and able to speak English and gave written informed consent in English.

### Gathering data

Family meetings were facilitated according to the model described by Murphy and took place in a location chosen by the patient [32]. A summary of the main features of this model is shown in Table 1. These family meetings were facilitated by the first author who has training and experience in grief and palliative care counselling, spiritual care, working with family groups and had completed a training program with the author of the model. The meetings were not recorded because it was considered that this could be uncomfortable for the participants. A genogram was constructed for each participating family. At the conclusion of the family meeting all individuals present were invited to make an appointment for a one on one in-depth interview about their experience of the meeting. These interviews (with 2 exceptions) took place within one week of the family meeting. Forty-seven interviews were conducted (11 patients and 36 family members).

**Table 1:** Key Features of the Murphy's Family Meeting Model

Aspect of Model	Main Features
**5 Part Paradigm**:	
1. Story of the Journey	- The patient speaks about their journey of illness including things that are and have been important to them and have helped them to make sense of it.
	- May also include the story of other important experiences and struggles of their life.

2. Worries and Fears	- The storyteller is encouraged to speak of their worries, fears and concerns about the illness and its outcomes.

3. Speaking of Roots	- Speaking of family history, recent and not so recent.
	- Allowing the pains and the joys to be openly expressed.

4. The Family Speaks	- Each person present has the opportunity to tell the story of their journey in relation to their loved one's illness
	- They speak of their history together as they have experienced it and have the opportunity to respond to what others have said.

5. The Closing or Blessing	- Bringing the meeting to a close in a manner appropriate for the family (may or may not include ritual with religious connotations).
	- It may be an opportunity for each person to say one thing that they value most about the person who is dying.

**The 3 Main Roles**:	

1. The Storyteller	- The one who speaks - everyone takes a turn (1 at a time)

2. Witness(es)	- The ones who listen preferably without judgement or interpretation - everyone else who is present

3. The Facilitator or Guide	- Supports, guides and moves the meeting along with the use of probe questions if necessary e.g. "Would you like to say more about that?" or "How did you feel about that?"

In depth interviews, which draw on an interpretive framework, were considered to be most appropriate for this study, not only because they provide rich data but because a number of researchers have concluded that this method is not excessively stressful for palliative patients and may be therapeutic, as it provides them with an opportunity to tell their story in their own way and at a pace they choose [43,44].

The interviews were audio recorded, with the written consent of the participants. Each interview began with the request that the participants tell the interviewer about their experience of the family meeting. Some prompt questions, such as; 'Would you like to tell me more about that?', 'What do you think will be the outcome of that?' and 'In what other ways was the family meeting helpful/not helpful to you?' were utilised to encourage in-depth reflection.

### Data analysis

All interviews were transcribed and, after checking for accuracy of transcription, were analysed with the assistance of the software package QSR NVivo 2.0 (QSR International, 2002). Ricoeur's theory of interpretation, which describes three levels of analysis, was utilized in the analysis of data in this study [37,45].

The first level of textual analysis, as proposed by this theory is that of considering only the internal nature of the text. In this process the text is seen as having no context and no author or audience. What arises from this is an 'explanation' based solely on a literal consideration of what the text says. Level two analysis begins the process of understanding what the text is talking about. This moves the analysis into the area of interpretation, as opposed to explanation, and its outcome is what Ricoeur describes as a naïve understanding of the text [37]. The outcome of level three data analyses is an appropriation, or making your own in-depth understanding of what the text is talking about. This is a process of moving back and forth between explanation and understanding. The acts of interpretation that are part of this process are informed by the knowledge, experience and beliefs that the researcher brings to the task. In this case the main researcher has a background of teaching, counselling and pastoral care within palliative care and other settings. A more detailed account of the analysis process is described in Tan, Wilson and Olver [42].

### Rigour and accountability

While more than one guide to rigorous reporting of research founded in hermeneutic phenomenology has been published, the one outlined by Rice and Ezzy was utilized in this study [39]. They describe five main areas of consideration to ensure rigour which are: theoretical (having a theoretical underpinning and methods which are consistent with this), procedural, interpretive, evaluative and reflexive rigour. Steps taken in this study to maintain rigour included: the appropriate choice of philosophical underpinning to the study and the selection of methods and sampling processes which were both consistent with this underpinning and suitable to provide the information sought, the documentation of all procedural and interpretive processes and decisions, taking into account ethical issues and transparency in accounting for the role of the researcher and its possible impact on the outcomes of the study.

As discussed in the basis for selecting Heideggarian inspired hermeneutic phenomenology for this study, it is not possible to produce a totally objective interpretation as there is no one 'true' interpretation of data. Hence the use of inter-rater reliability in coding (having two or more people code the same data and check correlation of coding) does not guarantee the reliability or validity of interpretations and so was not used in this study [39]. A more detailed account of measures taken to ensure rigour has already been reported elsewhere [42].

## Results

### Participants

A total of 66 patients were referred and received information sheets and where appropriate, consent forms and a short demographic questionnaire. Seven (11%) of those referred were not approached due to death (4) or sudden deterioration (3). Two (3%) who had been approached and agreed to participate subsequently deteriorated and did not take part in a family meeting. Forty-five (68%) of those referred declined to participate, their reason most commonly being that either they thought their family would not want to be involved or their family thought that participation would be too demanding for the patient. Twelve patients, (18%) of those referred (mean age 66.7) took part in a family meeting and their demographic details are presented in Table 2. A more detailed account of the challenges encountered in the recruiting process is given in Tan, Wilson, Olver and Barton [46].

**Table 2:** Patient Demographic Details

Age	Gender	Diagnosis	Religion	Place of Care
60	M	Liver cancer	Christian/Lutheran	Home

61	F	Cervical cancer	Christian	Home

76	M	Muscular Dystrophy	Christian/Congregational	Home

59	F	Wide spread cancer	Christian	Home

63	M	Pancreatic cancer	Christian	Home

76	F	Wide spread cancer	Christian	Hospice

60	F	Brain tumour	none	Hospice

59	F	Cancer (lung & bone metastases.	none	Hospice

82	F	Intra-peritoneal cancer	Christian	Hospice

73	M	Prostate cancer	Christian/Lutheran	Hospital

82	F	Breast cancer	Christian/Uniting Church	Home

73	M	Multiple Myeloma	none	Home

The age of family members, two-thirds of whom were female, ranged from 21-81 years (mean 50.5). The number of participants attending family meetings ranged from 2-11. Genograms and family profiles were created for each participating family.

### Key Themes

The seven themes, along with sub-themes and categories which were identified in the analysis of patient and family member interviews, as well as relevant researcher field notes, are tabulated in Table 3. These data were also categorised indicating whether they were predominantly relating to spiritual matters, psychosocial matters or both in the light of the definitions provided and taking into account the context of the family meeting and what was known of the participants and their backgrounds. They are illustrated below with direct quotes from the interview texts.

**Table 3:** Themes, sub-themes and categories

Theme	Sub-themes	Category/Spiritual	Category/Psychosocial
Personal experience of the meeting	The experience of speaking	experience of review *(meaning/purpose) *experience of being open *(deepening of relationship which goes beyond social engagement)*	topics covered/inhibitors to openness/experience of being open/value of speaking in group *(communication, social well-being)*

	How they felt		perceived feelings/getting realistic *(emotional wellbeing)*

	New understandings	of self/family members/family unit *(deepening of relationship which goes beyond social engagement)*	of self/family members/family unit *(communication, social well-being)*

Personal outcomes	Freedom to speak	said things would not have said/freer *(deepening of relationship which goes beyond social engagement)*	said things would not have said/freer *(communication)*

	Personal changes	specific changes eg faith, closure past hurts & guilt *(relationship to higher power, meaning)*	Talking more openly with a child *(communication)*

	Make a contribution	help research/other families/self/own family *(finding meaning & purpose)*	

Observations of others' experience	Openness	conservative but loosened up/courage to be open/as open as they could be *(for some deepening of relationship which goes beyond social engagement)*	conservative but loosened up/courage to be open/as open as they could be *(communication & social well-being)*

	Feelings		comfortable/emotional/painful *(emotional well-being)*

	New understanding	they understand better/learnt a lot *(for some deepening of relationship which goes beyond social engagement)*	they understand better/learnt a lot *(social well-being)*

	Other comment		definite benefit/initially sceptical *(social well-being)*

Observations of experience & out-comes for family unit	Impact on speaking together in future	reminded of the value of speaking more openly *(for some deepening of relationship which goes beyond social engagement)*	reminded of the value of this/broke the ice/sense of achievement/no change *(social well-being)*

	Impact on relationships	new ways of being together/new awareness/understanding *(for some deepening of relationship which goes beyond social engagement)*	strengthened family bond/new ways of being together/new awareness/understanding/reunion (*social well-being)*

	Impact on feelings		more comfortable together/overall felt better *(emotional well-being)*

	Impact on grieving process	may make it easier/give a different focus *(meaning)*	may make it easier/give a different focus *(emotional well-being)*

Meeting facilitation	Experience of facilitation		how they felt about it/impact of facilitation *(social & emotional well-being)*

	Qualities needed for facilitation	general qualities/handling problems/'outsider' *(for some deepening of relationship which goes beyond social engagement)*	general qualities/handling problems/'outsider' *(social & emotional well-being)*

How it could have been different	Who was present	Number/other family members/children *(potential impact)*	Number/other family members/children *(potential impact)*

	More meetings	Yes/doubtful *(potential impact)*	Yes/doubtful *(potential impact)*

	Meeting timing	Earlier/later/just right *(potential impact)*	Earlier/later/just right *(potential impact)*

	Needs not met by meeting	Need to talk 1 on 1/things couldn't say at meeting *(potential impact)*	Need to talk 1 on 1/things couldn't say at meeting *(potential impact)*

	Other meeting aspects	Place/style/hear more from others *(potential impact)*	Place/style/hear more from others *(potential impact)*

General applicability of the family meeting	Who would benefit	everyone/only in special circumstances/not for all *(potential impact)*	everyone/only in special circumstances/not for all *(potential impact)*

	Promoting it	general comments/specific means *(potential impact)*	general comments/specific means *(potential impact)*

#### THEME 1 - Personal experience of the meeting

Participants appreciated that 'there was enough opportunity to bring up whatever you wanted' but also that they had not felt undue pressure to discuss topics they preferred to avoid.

##### Spiritual Impact

Remembering the good times together gave them 'a lot of joy and comfort' but some also were reluctant to 'speak about death'. One family had a very strong culture that lacked openness in relation to sensitive issues, especially talking about matters related to death.

I think reminiscing is a great comfort at this stage, a great comfort (Patient 3par.19)

I think he (the patient) is just fantastic and just to blurt out things like that (about death), I don't know, I didn't think it was sort of appropriate at the time (Fanily5Member F para.48).

I thought 'Oh well I've done something in my life. (Patient5para247)

##### Psychosocial Impact

Although the 'relaxed atmosphere enabled openness' some had found that the experience of speaking in the family group about these matters was 'not as easy as you might think'.

A range of feelings were experienced by participants during the family meetings, which they verbalised during the interviews. These included feeling 'positive', 'relaxed', 'comfortable', 'surprised', 'less threatening than expected', 'a little nervous', 'emotional' and 'glad that no-one was out of control'. For a number of participants it was an opportunity 'to get real' about the situation.

I felt extremely comfortable with it. (Patient1para.13)

So I needed to remind myself that what my father was saying was very unusual for him in that meeting and I'm pleased to have heard it (Family3Member B para.23).

##### Both spiritual and psychosocial impact

Participants indicated that the family meeting had resulted in new understanding of themselves, of other individual family members and of the family as a unit. These new understandings included things such as 'I had thought what I did was just ordinary', 'I didn't know they cared', 'good to know how others feel' and 'we understand each other better now'.

Firstly, I think at least I know where I stand now with the family. That's something that I was struggling with for quite a while. So now it is a bit more clarified for me and I think that's great (Family1Member D para.5).

I didn't think they were so caring; the family was so caring (Patient5para9)

I thought that getting together with others and talking about it all together was probably a good thing because we all knew what was going on but no one had sort of said anything to the other about it (Family11MemberD para.23).

#### THEME 2 - Personal outcomes

##### Spiritual Impact

A few participants identified specific personal changes that had occurred for them as a result of the meeting. These included becoming '*re-orientated to Father in heaven' (Patient 3para10)*, becoming motivated to initiate contact with an estranged grandchild and closure of a past hurt. The sense of making a contribution to research, to other families or to their own family was an important motivating force for more than half of the participants.

It does sort of give you a feeling of having some sort of contribution to what is a very traumatic time and having some sort of thought that you might be making things better or easier or different for someone else going through the same experience so I think that's a positive (Family2MemberB para.74).

I felt very privileged yesterday that you were interested enough in us to do this, to giver us this opportunity.(Patient6para23

##### Psychosocial Impact

One young mother changed her view about talking to a child about death and funerals. Two participants on the other hand specifically commented that they didn't think there would be any changes for them.

I can remember my mother saying 'funerals are not for children' and she didn't believe in crying in front of people even and then I thought - I wasn't going to take my two year old daughter and now I think well maybe I will. I'll tell her what has happened of course to uncle because she understands everything and I think people underestimate children (Family5MemberE para.57).

##### Both spiritual and psychosocial impact

One of the personal outcomes of the family meeting for some of the participants was a greater freedom to speak. This was expressed from the perspective that they had been given an arena 'to say things that they would not have otherwise said' and it also manifest in a belief that 'it will be easier to talk about these things now' than it had been prior to the meeting.

I think it brings out a lot of little things that people don't say. Don't think to say sometimes (Family12MemberA para.6).

What you did has provided us with an arena to speak that would not have been available to the family any other way. (Patient3para25)

#### THEME 3 - Observation of others' experience

##### Psychosocial Impact

Feelings observed in others were also mostly seen as positive in nature and included 'supported and reassured', 'happy', 'more at ease' and 'calmed down'.

I think that was a really good relaxed environment for Dad to speak about his emotions because he doesn't really vocalise what he is feeling a lot. It benefited him in that respect, that he was in an environment that he felt comfortable opening up in and it wasn't done like my brother (who is a psychologist) would have done it which would have been really analytical and quite a bit more clinical I guess (Family1MemberC para.7).

A few participants reported noticing 'sadness' and 'stress' in others.

I looked at him when everyone was crying, and he looked very sad (Family5MemberA para.36).

##### Both spiritual and psychosocial impact

Generally the participants were surprised by and appreciative of the degree of openness of other family members during the meeting, only two indicating that they had hoped for greater openness.

I was wondering because Dad is not entirely conscious any more - I was wondering if something might slip out - even a veiled acknowledgement of difficulties we've had in the past or something but that didn't happen (Family3MemberB para.13).

I found it so interesting that she (daughter in law) who previously seemed outside the family had so much courage to open up. (Patient1para5)

The development of new understandings was also a strong feature in the participants' observations of others. These are illustrated in the following quotes: 'it was just nice for her to hear about how valued she is', 'it made her realise that it is no good sweeping it under the cupboard' and 'he would have learnt new things about the rest of the family'. Other more general comments included the observation that despite initial scepticism some had found a lot of benefit in the experience.

I can imagine it would have been quite eye-opening for him to hear of the rest of the family's perceptions and feeling on life over the last seven months. (Family1MemberB para.49)

I learnt a lot from it (Patient11para71)

#### THEME 4 - Observations of experience and outcomes for the family unit

The experience of the family meeting was perceived to have had an impact on some areas of family life.

##### Both spiritual and psychosocial impact

Many participants believed that 'family bonds had been strengthened' and this included 'feeling more comfortable talking about these things', finding 'their place in the family' and realising 'that we are not alone in this.' A number of participants anticipated that the experience of the family meeting opened things up more, 'may make the grieving process a little easier' or would at least 'give it a different focus', in this case a shift from trying to pretend it didn't happen to being able to celebrate the life of the deceased relative.

I feel more was said yesterday that hasn't been said before, especially when we touched on my parents in law's relationship (Family6MemberB para.7).

There was also an appreciation of the possibility that this increased openness could be experienced in a location and environment which most family members really valued.

I think we can get together again soon and talk about these things while we have a picnic down the river which most of us love. (Family5MemberF para.42)

I found that last night I was lying awake and thinking of some of my friends, one in particular, who died of cancer I suppose 25 years ago and she died in hospital and she had a really good marriage but her husband could not bear to come and see her or talk to her and she was lying there crying and crying because he couldn't talk to her before she died. I though weren't we lucky that we had yesterday (Patient6para13)

#### THEME 5 - Meeting Facilitation

A majority of the participants commented on their experience of the meeting facilitation.

##### Psychosocial Impact

Of particular importance to them was the 'informal', 'relaxed' and 'un-pressured' approach that made speaking about sensitive issues easier for them. They found that 'open prompts encouraged a wide ranging discussion' and that 'the opportunity for all to speak with minimal interruption' had been a very positive experience.

To see her now and the way she responds to not only just me, because I've got this illness, but to other members of the family, I think it is just wonderful (Patient1para.9).

##### Both spiritual and psychosocial impact

The participants appreciated the 'tactful handling of problem areas' and allowing the 'patient to set the scene'. A few family members considered that it was easier having a facilitator from outside the family. Only two problems were reported which were: frustration with a member who they considered 'needed gagging' and another who found some of the silences 'uncomfortably long'.

A number of the participants also commented on specific qualities that they had either observed in the facilitator of the family meeting, or that they thought would be important for facilitators of such meetings to demonstrate. These included: the ability to 'draw people out', 'to make people feel relaxed', to be 'perceptive', 'to handle sensitive issues' and to be 'adaptable to different families'.

I think it was interesting from the aspect of people actually verbalising things that they were going to miss about Mum. I think that was nice, so yes (Family2memberB para.15).

Very gentle, yes, and I think that is the best way to approach it. (Patient6para71)

#### THEME 6 - How it could have been different

##### Both spiritual and psychosocial impact

The main comment relating to the theme "how it could have been different" was the desire for, or interest in, having more than one such family meeting, considering that they 'would have been more relaxed' or 'would have had other issues they wanted to pursue'. Other comments related to the number of people present, 'I wish X was there' or to whether or not children should be involved, opinion being divided among participants who commented on this issue.

Definitely would have been value in other family members being present but then there may have been others who would have found it a problem fitting in (Patient3 para.39).

The timing of the meeting in relation to the illness trajectory and the place and style of the meeting were also matters raised by a small number of participants (only 1 in each case).

I suppose if it was done maybe a week or so earlier it might have made a difference. We need more time to ponder things (Family9MemberC para.14)

#### THEME 7 - General applicability of the family meeting

##### Both spiritual and psychosocial impact

It was widely agreed that a family meeting of this type 'should be available to everyone' and that it was valuable 'even if it was tense'. Many participants however acknowledged that 'some can't face it' or would find 'it just too hard' or that 'there would be different cultural issues'. The importance of 'respecting the patient's wishes' was stressed.

I think, I mean depending on the family, but I honestly think only a very distant family, a family that's got some real sort of issues I guess you could say would be the only ones that don't benefit from it (Family1memberD para.30).

Some participants also made suggestions for the promotion of these family meetings within the palliative care service and particularly that 'clear simple' information that promoted it 'as something normal' in the service, was important.

But I think if - like it was listed in the list of things like x-rays as a necessity well people would go along with it and if they didn't like it well they'd say 'Well we're not coming to another one' but if they all had to go to one at least it might start them thinking. Even if they said 'No, not going there again' (Family11MemberG para.125).

## Discussion

In exploring participants' experiences of the family meeting, seven dominant themes were identified. Within these themes frequent reference was made to most characteristics of the domain of spirituality as described in this study, which were a web of relationship with places, things, ourselves, significant others, a power beyond ourselves and the significance of these relationships in the finding of meaning and purpose. The family meeting also enabled a process of review which was appreciated by participants and confirms the findings of other studies which not only found that life review was an important process for those near end of life, but could also be instrumental in the finding of meaning and purpose [47].

Significant relationship with places and things did not, however, feature strongly in the majority of participants' described experience of the family meeting. One reference to this was made by a participant who was planning a future family picnic "in a place most of us love" and indicating the importance of this environment in relaxing and encouraging participation in conversation about matters previously not discussed by this family.

The main focus of most participants' experience of the family meeting was concerned with relationship with themselves and with significant others. Examples of these are numerous in themes one to four described above, although they are also implied in some aspects of themes five to seven. References to 'getting real about it', new understanding of self and others (theme1), initiating contact with estranged relatives and letting go of past hurts (theme 2), new degree of openness, new understandings (theme 3), strengthening of bonds, allowing grief to have a different focus (theme 4), changed experience of relationship (theme 5), a desire to have another meeting so others not present can also experience this (theme 6) are all examples of how the meeting contributed to strengthening the web of relationship with self and significant others for the participants of the family meetings.

Three participating families were quite open about their religious affiliations and regular participation in the life of a religious community, although only one participant made direct reference to this as an outcome of the meeting experience when they said that it had reconnected them to 'God the Father'. Murphy's family meeting model is however flexible enough to allow open discussion of religious issues and also to incorporate religious practices into its expression, especially, for example in the closing of the meeting which could include prayer, readings, lighting of candles and blessings if these are consistent with the practices and desires of the family involved.

The concept of finding meaning and purpose was also directly mentioned in several themes as an outcome of the family meeting experience. Two examples are: making a contribution to research, other families and our family (theme 2) and a person finally understanding how much they are valued (theme 5). There were however numerous examples where the adding of value and meaning were implied in the descriptions of participants' experience.

It is evident that significant psychosocial issues were also addressed in these family meetings. Communication within the family was identified especially in the first three of the seven themes which emerged in the data. Considerable emphasis was placed on greater than normal openness of family members and the opportunity this provided for people to connect with each other in a stronger more meaningful way. Good, clear, open communication has been acknowledged as vital in overcoming "conspiracies of silence" relating both to information about the illness and the care needed but also in overcoming built in family barriers to real connection with each other [31]. The realisation of 'not being alone' and that others 'think much more highly of you than you think' are very important contributors to social and mental well-being and self esteem.

Although not specifically identified by participants, it became apparent as interviews progressed, that the needs of some individuals to speak one on one with another person had not been met by the family meeting. There were issues that some participants couldn't or wouldn't raise in front of the family, but also others that arose afterwards having been stimulated by the meeting. The use this family meeting model as a means of providing spiritual care would not eliminate the need for some people to have the opportunity for more individual care.

It is also important to consider how broadly Murphy's model could be applied as an instrument for spiritual and psychosocial care [32]. Genograms and family profiles for each family were created utilizing the available data, but it was not possible to draw conclusions from these about the relationship, if any, between individual or family characteristics and outcomes of the family meeting. However, there are some observations worthy of note.

The findings suggest that a family's expressed view of their closeness and degree of openness are not reliable guides to the likelihood of members experiencing greater than normal degrees of openness in themselves or others during the family meeting, or to whether they are likely to come to new understandings as a result of the meeting. This is consistent with the concept of positive self-presentation and impression management [48]. Although, in our study there was a range of families from the perspective of socio economic status, religious expression and evidence of family conflict, there appeared to be no relationship between these factors and positive outcomes from the family meeting as they have been described.

The question then arises, 'Could there be a reliable way of determining which families would most benefit from this family meeting which works with a very subjective area of care, and which ones are most likely to decline to participate?' It is possible that the family relationships index may be a useful screening tool to identify those most likely to benefit [23]. Ethical requirements did not allow any pursuit of reasons for declining to participate, however, on the basis of the work of Kissane and Bloch it can be surmised that poor family communication, low cohesiveness, low levels of emotional expression and family conflict may have been contributing factors to the decision not to participate in our study, in some cases [23]. However definite conclusions cannot be drawn from our findings in this regard.

The differing environmental factors which may have impacted on the outcomes of this study are the location of the patient at the time of referral (home/hospital/hospice) and the cultural environments of the two palliative care service providers. This latter factor has been discussed in Tan, Wilson, Olver and Barton [46].

Patients who were at home at the time of referral were twice as likely to agree to participate, as those who were in a hospice, and about three times more likely to participate than those who were in hospital. Five of the participating patients are known to have died within two weeks of the family meeting intervention, three of these being in a hospice at the time of referral and two at home. These factors, although certainly not conclusive, suggest the possibility that willingness to participate may be improved for patients living at home, contrary to the results of another study, but would seem to be unrelated to imminent death [49].

Other circumstances which may have impacted on the outcomes, apart from family characteristics already discussed are; the nature of the illness (malignant or non-malignant), the time since diagnosis and the prognosis of life expectancy from the perspective of the patient and family members. Apart from a few comments about the timing of the meeting in relation to illness trajectory, this study did not provide data relating to these circumstances.

## Conclusions

In conclusion, it was found that for the participants of this study, the family meeting intervention was generally a positive experience and provided opportunity to explore and further enrich their spirituality as they expressed it as well as their psychosocial well-being. However, further research with more heterogeneous groups could be undertaken to consider the appropriateness and efficacy of Murphy's family meeting model for participants of differing cultural, religious and language backgrounds, different age groups (especially younger people) and the longer term benefits of the utilization of this model. This would certainly be facilitated by a preliminary study designed to identify or develop a suitable screening tool to effectively identify families most likely to benefit from this family meeting. This could be enhanced by a multisite application of the meeting model allowing application to a wider population. The impact of meetings over a longer period of time, for example 1 month, 3 months and 6 months post meeting, recognising that patients would often not be able to participate in these follow ups, would be beneficial in determining the longer term impact for families.

## Competing interests

The authors declare that they have no competing interests.

## Authors' contributions

HT conceived of the study, participated in the design of the study, did the recruiting, facilitated the family meetings, conducted the interviews, participated in data analysis and drafted the paper. AW contributed to the design of the study, the analysis of the data and construct of the paper. IO gave advice on recruiting procedures in the medical setting, participated in the analysis of the data and reviewed the draft of the paper. CB participated in the analysis of the data and reviewed the draft of the paper. All authors read and approved the final manuscript.

## Pre-publication history

The pre-publication history for this paper can be accessed here:

http://www.biomedcentral.com/1472-684X/10/7/prepub
